# Complementary Use of Shotgun Metagenomics and Multiplex PCR for Pathogen Detection on High-Touch Surfaces

**DOI:** 10.3390/microorganisms14102233

**Published:** 2026-10-03

**Authors:** Mohamad Taisir Ahmad Ghiba, Areej Alsheikh-Hussain, Turki Essa Alzahrani, Monther Alhamdoosh, Abdulkarim Fahad Alhetheel, Atif Hanif, Saleh Ahmed Eifan

**Affiliations:** 1 Botany and Microbiology Department, College of Science, King Saud University, Riyadh 11451, Saudi Arabia; 2 Computational Medicine & Bioinformatics Department, Genomic Medicine Centre of Excellence, King Faisal Specialist Hospital & Research Centre, Riyadh 12713, Saudi Arabiamalhamdoosh@kfshrc.edu.sa (M.A.); 3 Department of Pathology and Laboratory Medicine, College of Medicine, King Saud University, Riyadh 11451, Saudi Arabia

**Keywords:** environmental surveillance, shotgun metagenomics, QIAstat-Dx, multiplex PCR, fomites, SARS-CoV-2, viral enrichment

## Abstract

Shotgun metagenomics is being increasingly promoted as a pathogen-agnostic alternative to targeted PCR for environmental surveillance, yet its practical value on dry, low-biomass, host-dominated surfaces remains uncertain. In a companion study, we reported QIAstat-Dx multiplex PCR screening of 55 pooled high-touch surface samples from Riyadh, identifying 19 positive pools (34.5%). Here, in a retrospective exploratory comparison, we use those previously reported PCR calls as the targeted comparator and ask what unenriched shotgun metagenomic sequencing adds. Ten archived pools, each with at least one PCR-detected viral target, were sequenced (Illumina NovaSeq X Plus, 2 × 150 bp). Human-mapped reads constituted a substantial fraction of every library (mean 59.1%; range 42.1–80.2%). Among 14 evaluable PCR-positive target events, metagenomics assigned at least one read pair to the corresponding target taxon in 10 (71.4%; exact 95% CI, 41.9–91.6%). Because the cohort was preselected for viral PCR positivity, this value represents positive agreement/taxonomic concordance within the selected cohort and not diagnostic sensitivity. At the same ≥1 read-pair threshold, target taxa were also assigned to 91 of 246 (37.0%) evaluable pool-by-target cells in which PCR was negative; raising the threshold to ≥10 read pairs reduced positive agreement to 35.7% while background assignment remained 24.8%. Of six target categories with PCR-positive events, only coronavirus 229E was assigned more read pairs in its PCR-positive pool than in any PCR-negative pool. Across all ten pools, the three respiratory RNA-virus taxa represented among the PCR-positive calls accounted for only 105 classified read pairs in total—including assignments in both PCR-positive and PCR-negative pool-target cells—equivalent to 0.12% of the viral fraction. Even the broadest viral reference coverage was limited, as adenovirus in HP10 reached 16.39% (23 distinct alignment spans), while SARS-CoV-2 in CP17 reached 8.83% (12 spans); these partial, discontinuous alignments did not support robust whole-genome consensus reconstruction or genome-wide variant or phylogenetic analysis. Metagenomics nevertheless provided a broader taxonomic context beyond the targeted panel. Under this retrospective unenriched workflow, target recovery was variable, no tested assignment threshold separated a PCR-positive target signal from the background assignment, and shotgun metagenomics added ecological context rather than providing a reliable replacement for targeted multiplex PCR in this dataset.

## 1. Introduction

For most of the 20th century, environmental and clinical surveillance for infectious diseases relied on culture-based methods, which required pathogens to be grown in the laboratory prior to identification. Although culture remains important because of its specificity and ability to support downstream phenotyping (e.g., antimicrobial susceptibility testing), it is slow, labour-intensive, and biassed toward organisms that are readily culturable under standard conditions [1,2]. These limitations, combined with the need to diagnose complex, polymicrobial, or unexplained infections, drove a broad shift toward molecular and sequencing-based approaches in public health and clinical microbiology. Targeted nucleic-acid tests, such as PCR and multiplex panels, greatly increased speed and analytical sensitivity but still depend on prior knowledge of which pathogens to test for [3,4,5]. Against this backdrop, metagenomic next-generation sequencing (mNGS) emerged as an untargeted approach that can survey viral, bacterial, fungal, and parasitic nucleic acids without predefined primer sets [2,3,6,7]. Across selected clinical and environmental matrices, mNGS has broadened the detectable spectrum beyond predefined targeted assays and, when microbial abundance and coverage are sufficient, can support genome recovery for genomic surveillance and evolutionary analysis [1,2,4,8,9,10]. It has also contributed to the discovery and characterisation of emerging viruses and to wastewater metavirome studies of circulating viral diversity [4,8,11,12,13]. Accordingly, mNGS is increasingly incorporated into specialised clinical and public health workflows, but its performance remains strongly dependent on the specimen matrix, biomass, host background, controls, and analytical workflow [2,3,6,10,11,14].

Environmental metagenomics has delivered especially strong results in high-biomass matrices like wastewater, where dense viral and bacterial loads generate abundant microbial reads and robust assemblies, making mNGS highly effective for population-level pathogen surveillance. In contrast, dry fomites and high-touch surfaces represent an almost opposite scenario: they typically harbour low microbial biomass and are dominated by exogenous DNA from humans, dust, and the built environment, pushing sequencing toward the limits of detection [15,16,17,18,19,20]. Low-biomass samples are uniquely vulnerable to both environmental and reagent contamination because background DNA can easily overwhelm the true microbial signal, causing contaminants to dominate profiles and inflate apparent diversity [15,17,19,21,22,23,24]. Simultaneously, high-contact surfaces accumulate substantial host material (skin cells, sweat, mucosal droplets). In host-derived or host-proximal samples, even a small number of human cells can “completely inundate” microbial DNA; human reads routinely dominate metagenomes from skin and other body sites and can mask low-abundance pathogens unless aggressively depleted [25]. Benchmarks of metagenomic workflows have shown that even after experimental host-DNA reduction, up to 70–90% of reads may still map to the host in low-biomass or host-adjacent samples, forcing ultra-deep sequencing and complex decontamination pipelines to recover meaningful microbial data [25,26,27,28]. Consensus guidelines, therefore, single out low-biomass environments (air, certain human tissues, treated water, hyper-arid or cleaned surfaces) as requiring special protocols, dense negative controls, and cautious interpretation, because standard practices that work in faeces or wastewater can become misleading when the microbial load is near background levels [15,16,17,18,19,20]. Consequently, high-touch surfaces form a particularly challenging matrix for untargeted sequencing; they combine low microbial biomass, high host DNA, and extreme susceptibility to contamination, raising legitimate questions about how well mNGS performs for routine viral surveillance on dry fomites compared to its success in wastewater.

The objective of this study was to perform a retrospective exploratory comparison between a targeted molecular syndromic panel and untargeted metagenomic sequencing for respiratory and enteric pathogen targets on high-touch surfaces. The QIAstat-Dx Respiratory SARS-CoV-2 Panel and QIAstat-Dx Gastrointestinal Panel 2 provided the previously reported targeted comparator, whereas Illumina shotgun metagenomic sequencing provided an untargeted taxonomic survey [29,30,31,32]. Unlike prospective diagnostic-accuracy studies, the present analysis used archived pools selected after PCR screening and therefore was not designed to estimate diagnostic sensitivity or specificity. We focused instead on target-level positive agreement, background taxonomic assignment in PCR-negative pool-by-target cells, threshold dependence, genome-level support, and the broader taxonomic context recovered by sequencing. This framing allows the study to address the practical question of what unenriched shotgun metagenomics adds to an existing targeted environmental surveillance dataset in a low-biomass, host-dominated matrix.

This manuscript is a direct companion to our recently published environmental surveillance study of the same sample set [33], in which the QIAstat-Dx screening results used here as the comparator were first reported. The present work does not re-report that survey as a new dataset. Previously published PCR detections and Ct values are reproduced only where needed to define the comparator and attributed to the original publication. The sequencing data, taxonomic classifications, threshold analyses, background-assignment analyses, and genome-coverage interpretation reported here have not previously been published as a journal article; an earlier presentation of parts of the sequencing analysis was included in the first author’s doctoral dissertation at King Saud University in 2026.

## 2. Materials and Methods

### 2.1. Environmental Sampling and Processing

This was a retrospective secondary methodological analysis of archived material from the 55 pooled environmental samples described in the companion surveillance study [33]. Sampling was conducted across high-traffic zones in Riyadh between February 2023 and May 2024 using sterile flocked nylon swabs placed in viral transport medium, transported at 4 °C, and then stored at −80 °C, as previously reported [33]. Individual swabs had been combined into 55 pools according to the study’s setting- and sampling-period-specific pooling scheme (4–7 swabs per pool) [33]. The present sequencing analysis begins with the subset of 10 archived pools selected because each contained at least one previously reported viral QIAstat-Dx detection. The complete original surveillance dataset is available in the companion publication [33]; previously published PCR information is included in the present Supplementary Materials only where needed to document the comparator used for the sequencing analysis (Supplementary Tables S1–S3).

### 2.2. Targeted Multiplex PCR

The multiplex PCR screening used here as the targeted comparator was performed and reported previously [33], and it is described only to the extent needed to interpret the metagenomic comparison. All 55 pooled samples had been screened with the QIAstat-Dx^®^ Respiratory SARS-CoV-2 Panel and the QIAstat-Dx^®^ Gastrointestinal Panel 2 (QIAGEN, Hilden, Germany). Positivity followed the instrument’s qualitative call algorithms, and Ct values were retained to contextualise target-template abundance; Ct values > 35 are described as high-Ct/low-template events rather than as a separate diagnostic category. Internal controls (MS2 bacteriophage for the respiratory panel and *Schizosaccharomyces pombe* for the gastrointestinal panel) amplified successfully across all 110 cartridge runs, and no run was invalidated. These internal controls document cartridge-level process validity but are not substitutes for sequencing extraction blanks, library blanks, or positive spike controls.

### 2.3. Shotgun Metagenomic Sequencing

Total nucleic-acid extraction from the 10 selected archived pools was performed commercially by Azenta Life Sciences using the PureLink™ Viral RNA/DNA Mini Kit (Invitrogen, Thermo Fisher Scientific, Carlsbad, CA, USA). The Azenta order documentation identifies the service as Standard RNA-Seq with rRNA depletion, a target output of approximately 20 million read pairs per sample, with explicit instructions not to perform DNase treatment. Because DNase treatment was omitted, DNA present in the total nucleic-acid extract was not deliberately removed before library preparation. RNA was processed under the provider’s rRNA-depleted Standard RNA-Seq workflow. The provider QC form labelled CP18, HP2 and HP10 as “Viral Particles (dsDNA)” and the remaining seven pools as “Viral Particles (RNA)”. The three dsDNA-labelled pools were the same pools in which QIAstat-Dx detected adenovirus, a double-stranded DNA virus. Pre-library quality control included NanoDrop spectrophotometry, Qubit fluorometric assessment and Fragment Analyzer/ProSize analysis, with RNA quality reported as RQN and DV200. Across the ten pools, RQN ranged from 6.1 to 8.6 (median 7.35) and DV200 from 67.0 to 85.0; per-sample values and the provider QC record are reproduced in Supplementary Document S1. The available service records do not identify the exact commercial library-preparation kit or fully specify how retained DNA- and RNA-derived products were represented in the final library; these details are therefore not inferred. The final libraries were sequenced on an Illumina NovaSeq X Plus platform using a 25B flow cell in a paired-end 2 × 150 bp configuration. Provider sequencing statistics and the available QC records are reproduced in Supplementary Document S1.

### 2.4. Bioinformatics Analysis

Raw sequencing reads were processed using a custom bioinformatics pipeline designed for low-biomass environmental samples. Adapters and low-quality bases were removed from raw reads using fastp (v0.24.0) [34]. Quality metrics before and after trimming are summarised in Supplementary Document S1. We implemented a critical host-depletion step to remove anthropogenic contamination; trimmed reads were aligned to the human reference genome (GRCh38) using the bwa-mem2 aligner (v2.2.1) [35]. All reads mapping to the human genome were computationally discarded to isolate the environmental microbial signal.

The remaining unmapped reads were classified taxonomically using Kraken2 (v2.17.1) [36] against the Standard Plus Protozoa and Fungi database (k2_pluspf_20250714). A custom Python 3 mapping script linked Kraken2 taxonomic identifiers to QIAstat-Dx panel targets using a predefined lookup table. The analysis scripts, target mapping file and documentation are publicly available at https://github.com/malhamdoosh/SurfaceMetagenomicScripts (accessed on 22 September 2026). The predefined target list uses genus-level mappings for Enterovirus, Mastadenovirus, Rotavirus, Mamastrovirus and *Cryptosporidium*; other evaluable target mappings use species- or subspecies-level entries where available. Counts were aggregated without double-counting nested Kraken2 rows: where a genus/clade total subsumed species rows for the same panel target, the clade total was used and the nested rows were not added; where a QIAstat-Dx target represented genuinely distinct species (for example, *Campylobacter jejuni*, *C. coli* and *C. upsaliensis*), the relevant species counts were summed. A genus- or clade-level Kraken2 assignment was not upgraded to species-level identification in the absence of separate reference-alignment support. The aggregation rule for every target is provided in Supplementary Table S1 and in the public target_list.txt file. Because QIAstat-Dx is not an independently validated reference method for environmental surfaces, and because only virus-positive pools were selected for sequencing, the analysis is not a diagnostic-accuracy validation. Pool-by-target cells were classified neutrally as PCR+/mNGS+, PCR+/mNGS−, PCR−/mNGS+ or PCR−/mNGS−. Results are shown at exploratory thresholds of ≥1, ≥5 and ≥10 classified read pairs; a ≥1 read-pair assignment is treated as a permissive taxonomic signal and not as a validated detection threshold. Positive agreement is calculated only over evaluable PCR-positive target events, and 95% confidence intervals for all positive-agreement estimates were calculated using the exact Clopper–Pearson binomial method. Targets absent from the Kraken2 database (Parainfluenza viruses 1–3, *Cyclospora cayetanensis*, and *Entamoeba histolytica*) or not resolvable through taxonomy alone (EAEC, EPEC, ETEC, STEC, and EIEC, which require virulence-gene evidence) were designated not evaluable and excluded from the agreement denominator.

To assess whether Kraken2 assignments concordant with PCR were supported by reference alignment and to evaluate duplication, all 10 PCR+/mNGS+ target-pool combinations at the ≥1 read-pair threshold were re-examined. Target-assigned reads were extracted from the host-depleted paired FASTQ files and aligned with bwa-mem2 to a selected reference genome or assembly for the corresponding taxon; the reference accession or assembly and mapping level are listed in the Supplementary Materials. Reference breadth was calculated as the percentage of reference bases covered by at least one primary mapped alignment. Duplicate-aware support was summarised by collapsing complete mapped read pairs with identical unordered mate start coordinates into distinct mate-pair alignment spans; when only one mate mapped, unique mapped singleton loci were recorded separately. Without unique molecular identifiers, these span counts are not interpreted as exact counts of independent template molecules. For genus-level targets (Adenovirus, Rhinovirus/Enterovirus, and *Cryptosporidium*), alignment to a single representative species/reference was used only as an orthogonal support check and not as species-level confirmation. The public repository also documents the extraction, alignment, breadth, and deduplication workflow.

Per-sample read counts after each processing step are provided in Supplementary Document S1.

## 3. Results

### 3.1. Human-Mapped Read Burden in High-Touch Surface Samples

**Figure 1:**
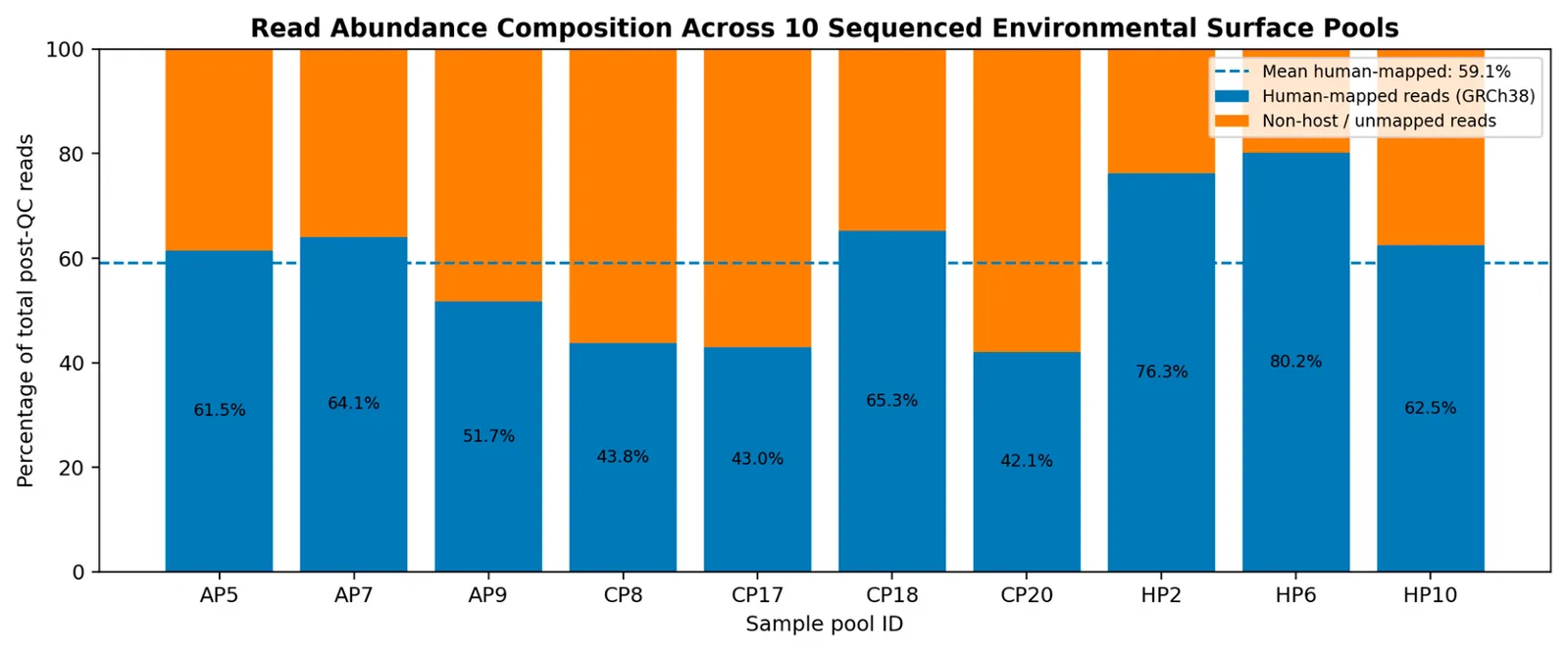
Read abundance composition across the 10 sequenced environmental surface pools. Stacked bars show the percentage of post-QC reads mapped to the human reference genome (GRCh38) and the remaining non-host/unmapped fraction. The mean human-mapped fraction was 59.1% (range 42.1–80.2%). Individual pool IDs are shown on the *x*-axis; the dashed horizontal line indicates the mean human-mapped-read percentage.

Human-mapped reads constituted a substantial fraction of all 10 sequenced surface pools, accounting for a mean of 59.1% of total reads (range 42.1–80.2%), as determined by alignment to the human reference genome (GRCh38) using bwa-mem2 (Figure 1). Individual pool percentages varied substantially. For example, HP6 showed 80.2% human reads (6.9 M non-human reads, equivalent to 3.5 M non-human read pairs, out of 35.0 M total reads after QC), while CP20 showed 42.1% human reads (22.6 M non-human reads, equivalent to 11.3 M non-human read pairs, out of 39.1 M total reads after QC). Conversely, the microbial/unmapped fraction ranged from 19.8% (HP6) to 57.9% (CP20), corresponding to 6.9 M–22.6 M non-human reads per pool. Host fractions are reported here from alignment to GRCh38 with bwa-mem2, which is the estimate used for host depletion throughout this study; the per-sample “% *Homo sapiens*” values shown in the MultiQC summary of Supplementary Document S1 are derived instead from k-mer classification of the unfiltered reads and are correspondingly lower. The two estimates are methodologically distinct and not directly comparable. These high host backgrounds substantially reduced the effective microbial library size available for pathogen detection.

Across the 10 pools, sequencing yielded a median of approximately 17.8 million read pairs (range 16.1–19.5 million); after human-read removal, a median of approximately 6.8 million non-human read pairs remained per pool (range 3.5–11.3 million; lowest in HP6, highest in CP20). This produced uneven effective non-human sequencing depth across the dataset, although the PCR target was still recovered in HP6—the lowest-depth pool. Per-pool sequencing and host-removal metrics are provided in Supplementary Table S4.

### 3.2. Concordance Between PCR and Shotgun Metagenomics

**Figure 2:**
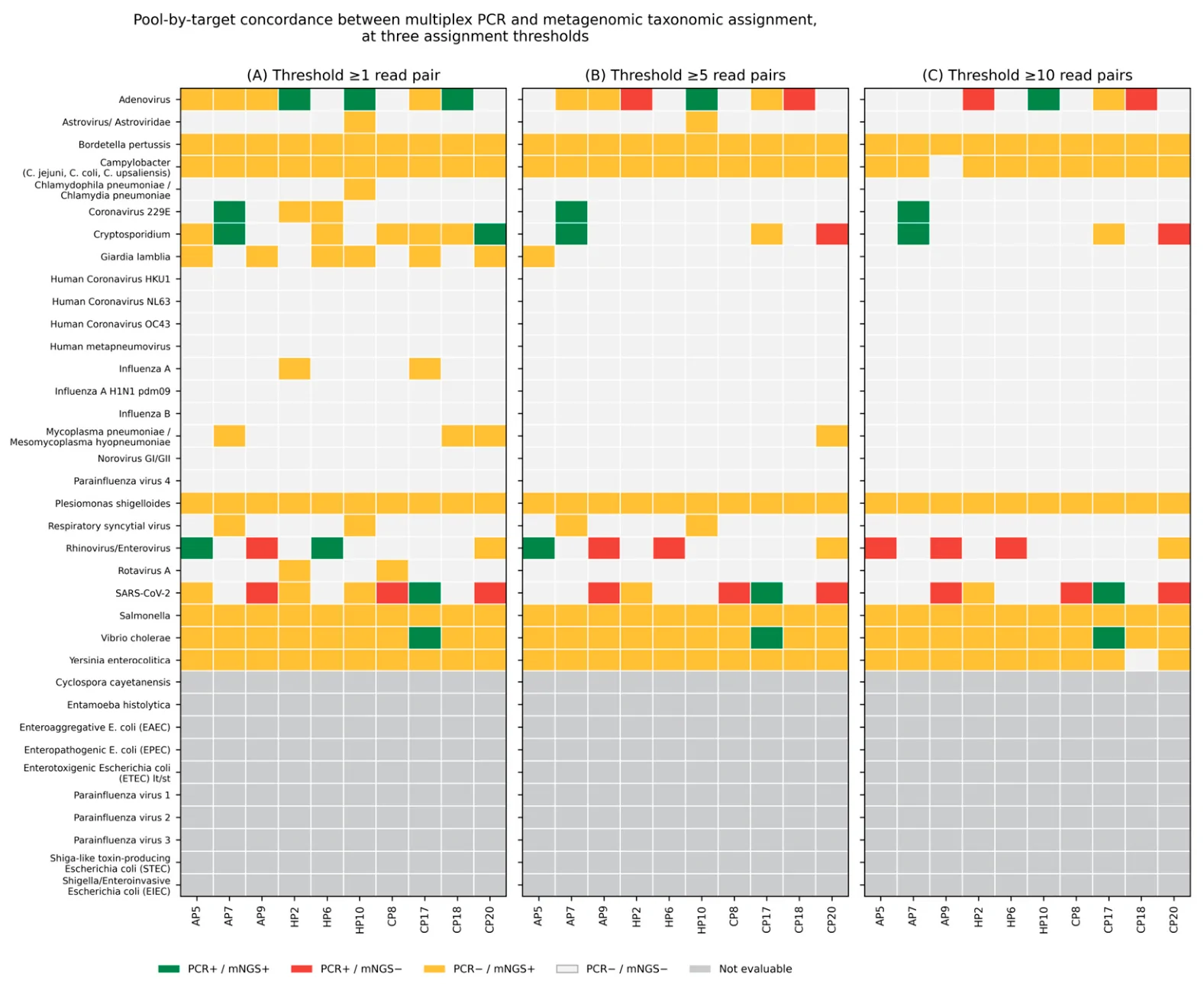
QIAstat-Dx/mNGS comparison across the 10 sequenced surface pools at three Kraken2 assignment thresholds. (**A**) Exploratory ≥ 1 read-pair threshold. (**B**) Intermediate ≥ 5 read-pair threshold. (**C**) More stringent ≥ 10 read-pair threshold. Rows represent QIAstat-Dx targets/pathogen categories and columns represent sample pools. Cell categories are PCR+/mNGS+, PCR+/mNGS−, PCR−/mNGS+, PCR−/mNGS−, or not evaluable. Targets absent from the Kraken2 database and *Escherichia coli* pathotypes requiring virulence-gene evidence are shown as not evaluable. Source data: Supplementary Table S1.

As previously reported for this sample set, QIAstat-Dx screening of the 55 pools identified 19 positive pools [33]; these PCR calls are used here only as the targeted comparator. The 10 archived pools selected for sequencing each contained at least one viral PCR-positive event, whereas pools positive only for non-viral targets were not sequenced. Within these 10 pools, there were 16 previously reported PCR-positive target events (11 viral and five non-viral). Two EAEC events (CP20 and HP10) were not evaluable by taxonomic classification because EAEC identification requires virulence-gene evidence, leaving 14 evaluable PCR-positive events. At the permissive ≥ 1 read-pair threshold, the corresponding target taxon was assigned in 10 out of 14 events (71.4%; exact 95% CI, 41.9–91.6%; Figure 2A). These comprised seven viral events—SARS-CoV-2 in CP17 (39 high-confidence read pairs [78 individual reads] after human-read filtering); Adenovirus in HP10, HP2, and CP18; Coronavirus 229E in AP7; and Rhinovirus/Enterovirus in AP5 and HP6—and three non-viral events: *Vibrio cholerae* in CP17 and *Cryptosporidium* in AP7 and CP20. Because the same taxonomic assignments also occurred in PCR-negative cells (Section 3.3), these are reported as assignments concordant with PCR calls rather than as independent confirmation. The four PCR+/mNGS− events were all viral: SARS-CoV-2 in AP9, CP8 and CP20, and Rhinovirus/Enterovirus in AP9. Several PCR+/mNGS+ events had only sparse support (for example, Adenovirus in HP2, four read pairs at Ct 36.2 [33]), underscoring the permissive nature of the ≥1 threshold. At ≥5 read pairs, positive agreement occurred in six out of 14 evaluable events (42.9%; exact 95% CI, 17.7–71.1%; Figure 2B), and at ≥10 read pairs, it decreased to five out of 14 (35.7%; exact 95% CI, 12.8–64.9%; Figure 2C). The corrected hierarchical aggregation for *Cryptosporidium* and Rhinovirus/Enterovirus did not change these threshold-level concordance totals. The complete pool-by-target matrix, including PCR call, Ct, Kraken2 read-pair count, evaluability, and category at all three thresholds, is provided in Supplementary Table S1. No enteric viral target was PCR-positive in the sequenced subset; low-level mNGS assignments to rotavirus and astrovirus occurred only in PCR-negative cells and are treated as exploratory background assignments rather than as detections.

### 3.3. Background Taxonomic Assignment in PCR-Negative Cells

Because only virus-positive pools were selected for sequencing, positive agreement above was calculated over PCR-positive cells alone. Examination of the PCR-negative cells shows that low-count taxonomic assignment was common outside cells in which PCR detected the corresponding target. Of 246 evaluable PCR-negative pool-by-target cells, 91 (37.0%) were assigned at least one read pair to the target taxon. *Vibrio cholerae* was assigned in all ten pools (156–1393 read pairs), with the highest count in HP10 (1393), a PCR-negative cell that exceeded the PCR-positive CP17 count (1251). *Cryptosporidium* reached 14 read pairs in PCR-negative CP17, equalling the count in PCR-positive AP7; Rhinovirus/Enterovirus reached 10 read pairs in PCR-negative CP20, exceeding both PCR-positive cells (AP5, seven; HP6, four). *Salmonella, Campylobacter*, *Yersinia enterocolitica*, *Plesiomonas shigelloides*, and *Bordetella pertussis* were assigned across all ten pools despite no PCR-positive event for those targets in the sequenced subset. Of the six target categories represented by at least one PCR-positive event, only coronavirus 229E was assigned more read pairs in its PCR-positive pool (AP7, 25) than in any PCR-negative pool (maximum of three). Raising the assignment threshold did not eliminate this background: at ≥5 read pairs, positive agreement was 42.9% (exact 95% CI, 17.7–71.1%) while background assignment remained 28.5%; at ≥10 read pairs, positive agreement was 35.7% (exact 95% CI, 12.8–64.9%) and background assignment remained 24.8%. Thus, no threshold tested separated PCR-positive target signal from background taxonomic assignment in this dataset. Per-target background rates and the complete threshold analysis are provided in Supplementary Table S1. The overall domain-level and viral-fraction composition of the libraries, which provides context for these background assignments, is summarised in Supplementary Table S5 and in Section 3.5.

### 3.4. Reference-Alignment and Duplicate-Aware Examination of Concordant Assignments

To determine whether a low-count Kraken2 assignment was supported by independent reference alignment, we examined the pool CP17 in detail. CP17 had previously tested positive by QIAstat-Dx for SARS-CoV-2 (Ct 33.9) and *Vibrio cholerae* (Ct 37.9) [33]. The SARS-CoV-2 event therefore provided the strongest available viral case for evaluating whether taxonomic assignment translated into geographically distributed reference-genome support.

**Figure 3:**
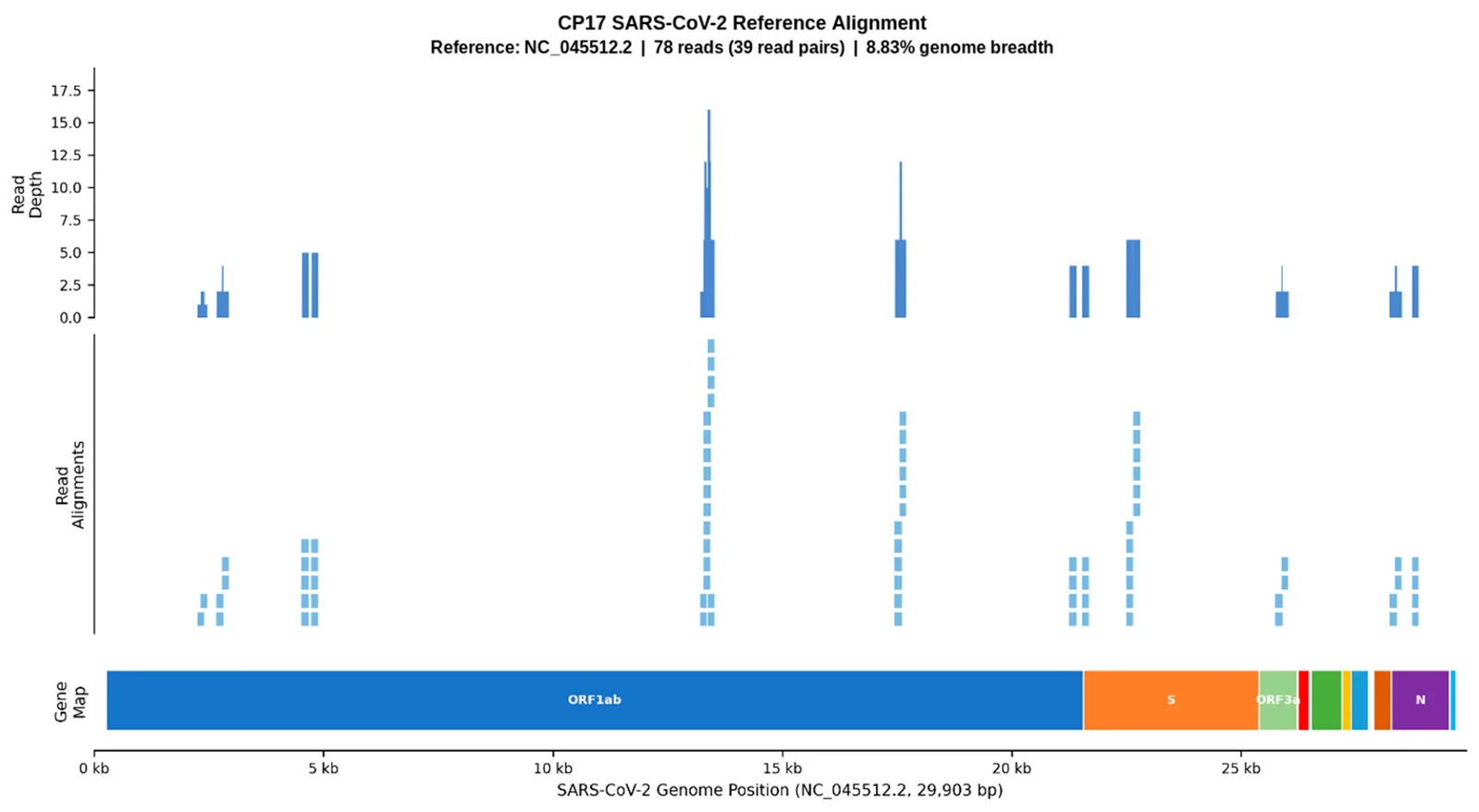
Alignment visualisation of CP17 SARS-CoV-2 reads mapped to the reference genome (NC_045512.2). Top panel—read depth per genomic position; middle panel—individual read alignments; bottom panel—SARS-CoV-2 gene map. Each light-blue bar in the middle panel represents one aligned read; coloured blocks in the gene map denote individual SARS-CoV-2 genes and open reading frames, and the smaller open reading frames between S and N are shown without labels for legibility. Only high-confidence reads (MAPQ 60) that remained after human-read filtering are shown. Read alignment was performed using bwa-mem2 (v2.2.1); the visualisation was generated using a custom Python 3 script (Matplotlib 3) from the resulting BAM file.

Initial Kraken2 classification before human-read removal assigned 212 read pairs to SARS-CoV-2. Alignment-based filtering against GRCh38 with bwa-mem2 showed that 173 of these pairs (81.6%) were human-derived sequences that had been taxonomically misassigned, leaving 39 high-confidence read pairs (78 individual reads) for SARS-CoV-2. Reference alignment to NC_045512.2 placed these reads in eight discontinuous clusters across ORF1ab (~2.3, ~4.5, ~13.2, ~17.4 and ~21.3 kb), S (~22.5 kb), ORF3a (~25.7 kb), and N (~28.2 kb), covering 8.83% of the genome (Figure 3). The 39 read pairs collapsed to 12 distinct mate-pair alignment spans, indicating substantial duplication; because no UMI-based deduplication was available, these spans cannot be interpreted as an exact count of independent biological template molecules. The mean depth over covered positions was 4.43×, but the coverage was sparse and discontinuous. The evidence was insufficient for reliable consensus reconstruction, variant analysis, lineage assignment or phylogenetic interpretation (Supplementary Table S6). This case illustrates why low-count k-mer assignments require orthogonal alignment validation before being interpreted as organism-level confirmation.

Extending the reference-alignment check to all 10 PCR+/mNGS+ target-pool combinations showed heterogeneous support (Supplementary Table S7). Four assignments had predominantly full-length mapped reads and broader support against the selected reference: Coronavirus 229E in AP7 (25 complete mapped read pairs, eight distinct mate-pair start spans, 7.00% breadth), SARS-CoV-2 in CP17 (39 pairs, 12 spans, 8.83% breadth), Adenovirus in HP10 (50 complete mapped pairs from 53 Kraken2-assigned pairs, 23 spans, 16.39% breadth against the selected Human adenovirus D reference), and *Vibrio cholerae* in CP17 (1114 complete mapped pairs from 1251 assigned pairs, 291 spans, 1.63% breadth across the two-chromosome reference). Two additional genus-level assignments produced only short partial support: *Cryptosporidium* in AP7 had 16 mapped individual reads but only two complete mapped pairs, one distinct mate-pair span plus three unique singleton loci, with 49 reference bases covered (0.00054% breadth) and a median aligned length of 43 bp; Rhinovirus/Enterovirus in HP6 had three mapped individual reads, no complete mapped pair, one unique singleton locus, 43 covered bases (0.58% breadth), and a median aligned length of 43 bp. Four sparse assignments—Rhinovirus/Enterovirus in AP5 (seven assigned pairs), Adenovirus in CP18 (three) and HP2 (four), and *Cryptosporidium* in CP20 (three)—yielded no reads aligning to the selected representative reference. Because Adenovirus, Rhinovirus/Enterovirus, and *Cryptosporidium* were compared at the genus level, the failure to align to one representative reference does not by itself invalidate the genus-level Kraken2 assignment; instead, it indicates a lack of orthogonal support against that specific reference.

Across the targets with mapped support, duplicate-aware span counts were substantially lower than the corresponding raw or complete mapped read-pair counts, indicating repeated coverage of the same alignment positions. This was most apparent for CP17 SARS-CoV-2 (39 assigned pairs to 12 distinct spans), AP7 Coronavirus 229E (25 to eight), HP10 Adenovirus (50 complete mapped pairs to 23 spans), and CP17 *V. cholerae* (1114 complete mapped pairs to 291 spans). These summaries support the concern that raw pair counts can overstate independent evidence. They are reported as alignment spans rather than molecular counts because unique molecular identifiers were not used.

CP17 also shows that the host fraction alone did not explain recovery. The pool had one of the lowest host backgrounds in the dataset (43.0%; only CP20 was lower) and the lowest SARS-CoV-2 Ct (33.9), yet it yielded only 39 high-confidence read pairs distributed across 12 distinct alignment spans and 8.83% genome breadth. Across the ten pools, the mean host fraction was actually higher in the seven pools in which the selected PCR target had a corresponding mNGS assignment (64.7%) than in the three without one (45.9%); HP6, with the highest host fraction (80.2%), retained its target assignment, whereas CP20, with the lowest host fraction and the same SARS-CoV-2 Ct as CP17, did not. These observations reinforce that recovery was variable and cannot be attributed to host fraction alone.

### 3.5. Broader Taxonomic Composition of the Shotgun Metagenomes

**Figure 4:**
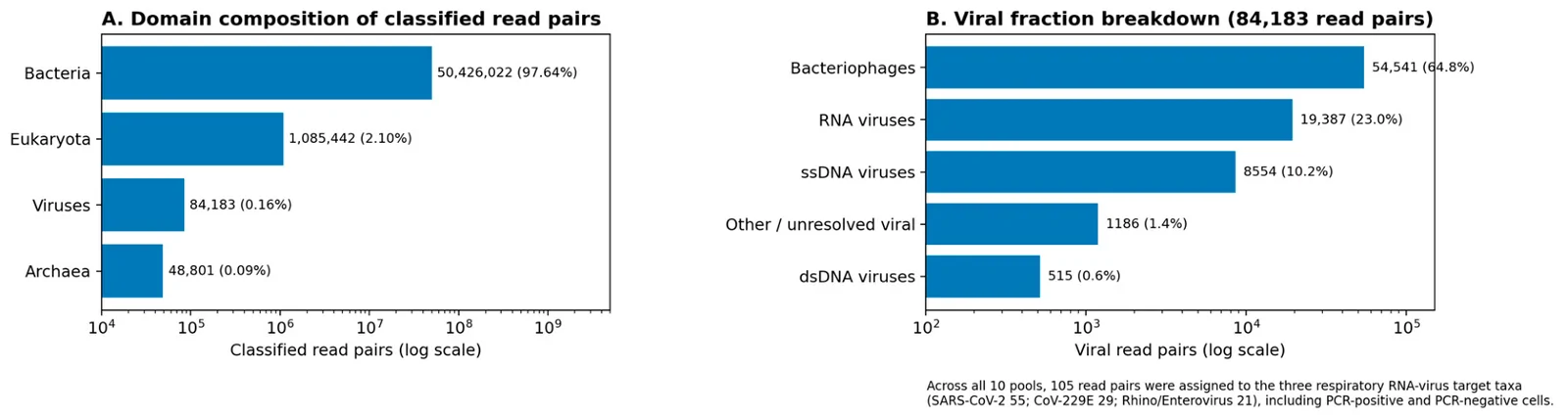
Shotgun-metagenomic composition of the 10 high-touch surface pools. (**A**) Domain-level composition of classified read pairs; viral assignments accounted for 0.16%. (**B**) Composition of the viral fraction (84,183 classified read pairs). Across all ten pools, the three respiratory RNA-virus taxa represented among PCR-positive calls accounted for 105 classified read pairs (0.12% of the viral fraction; approximately 2 read pairs per million classified read pairs), including assignments in both PCR-positive and PCR-negative pool-target cells. The figure therefore describes taxonomic composition, not independent confirmation of pathogen presence.

Beyond the targeted comparison, taxonomic classification of the host-depleted data described the broader taxonomic composition of the surface pools and the background against which target viruses were sought. Classified sequence units were overwhelmingly bacterial (50,426,022 classified read pairs; 97.6%), with smaller eukaryotic (1,085,442; 2.1%), archaeal (48,801; 0.09%) and viral (84,183; 0.16%) fractions; a further 21,100,500 read pairs were unclassified/no database match (Figure 4; Table 1). The most abundant taxa were human skin and oral commensals together with environmental organisms, including *Cutibacterium acnes* (6,568,114 read pairs), environmental *Bifidobacterium* (2,561,332), *Sphingobium yanoikuyae* (758,483), *Malassezia restricta* (672,093), *Corynebacterium tuberculostearicum* (636,464), and oral commensals, such as *Streptococcus mitis*, *Rothia*, *Kocuria*, and *Micrococcus* spp. This profile is consistent with repeatedly touched surfaces and represents the abundant background against which trace pathogen assignments were interpreted.

Within the small viral fraction, bacteriophages predominated (54,541 read pairs; 64.8%), principally *Cutibacterium/Propionibacterium* phages associated with the dominant skin bacteria. RNA viruses (19,387 read pairs; 23.0%) were dominated by plant viruses, chiefly tobamoviruses (tomato brown rugose fruit virus, 10,297 read pairs; tobacco mosaic virus, 4201 read pairs). Across all ten pools, the three respiratory RNA-virus taxa represented among the PCR-positive calls—SARS-CoV-2 (55 read pairs), coronavirus 229E (29), and rhinovirus/enterovirus (21)—accounted for 105 classified read pairs in total. This total includes assignments in both PCR-positive and PCR-negative pool-target cells and therefore is not a count of independently confirmed target events. In addition, ssDNA viruses accounted for 8554 read pairs (10.2%), including beta-papillomavirus/HPV-5 (4530) and adeno-associated virus 2/dependoparvovirus (~1500); meanwhile, other/unresolved viral assignments contributed 1186 read pairs (1.4%), and dsDNA viruses contributed 515 read pairs (0.6%), including Adenoviridae.

These off-panel assignments are reported as environmental or commensal context and are not interpreted as newly identified human pathogens. HPV-5 and adeno-associated virus are common constituents of the human skin virome, while tobamoviruses, environmental moulds (*Aspergillus* and *Penicillium* spp.), and *Saccharomyces cerevisiae* are compatible with environmental or dietary input (Table 2). A larger assignment was made to *Plasmodium relictum* (avian malaria; 113,953 read pairs); because this organism is not a human pathogen in this context and no orthogonal confirmation or negative sequencing controls were available, the signal is interpreted cautiously as possible environmental bird-associated material, sequence cross-reactivity, or a classification artefact. Parainfluenza virus 5/simian virus 5 (996 read pairs across unrelated pools) is likewise treated as a possible reagent- or database-associated signal rather than a confirmed environmental pathogen detection.

Per-pool counts for the QIAstat-Dx comparison targets are provided in Supplementary Table S1.

## 4. Discussion

### 4.1. Reduced Target Recovery and Background Assignment in Unenriched Metagenomics

At the permissive ≥ 1 read-pair threshold, shotgun metagenomics produced corresponding taxonomic assignments for seven out of 11 viral and all three evaluable non-viral PCR-positive events (10/14; 71.4% positive agreement; exact 95% CI, 41.9–91.6%). However, the same threshold assigned target taxa to 37.0% of evaluable PCR-negative cells, so these matches cannot be treated as independent confirmations of the PCR calls. Metagenomic respiratory-pathogen assignments in PCR-negative surveillance specimens have also been reported elsewhere [37], underscoring that discordant mNGS assignments are not intrinsically false but require orthogonal validation and appropriate controls. Viral support was generally sparse: adenovirus was assigned in HP10 (53 read pairs), HP2 (four) and CP18 (three), coronavirus 229E in AP7 (25), rhinovirus/enterovirus in AP5 (seven) and HP6 (four), and SARS-CoV-2 in CP17 (39 high-confidence read pairs after host filtering). The three evaluable non-viral PCR-positive events also had corresponding assignments—*Vibrio cholerae* in CP17 (1251 read pairs) and *Cryptosporidium* in AP7 (14) and CP20 (three)—but overlapping or higher counts in PCR-negative cells prevented organism-level confirmation from taxonomy alone. Taken together, the data show that unenriched sequencing can register panel-target taxa in this matrix, but low-count k-mer assignment does not reliably distinguish PCR-positive from PCR-negative pool-target cells under the thresholds tested.

The post hoc alignment analysis reinforced this caution. Of the ten PCR+/mNGS+ cells at the permissive threshold, four showed predominantly full-length support against the selected reference, two showed only short partial alignments, and four sparse genus-level assignments did not align to the selected representative reference (Supplementary Table S7). These findings were not used to retroactively redefine the primary Kraken2 concordance categories because reference choice can materially affect genus-level targets, but they show why low-count k-mer assignments require orthogonal confirmation before being treated as organism detection.

In the selected cohort, QIAstat-Dx had previously yielded positive target calls for several events for which unenriched Illumina shotgun sequencing produced zero or only sparse corresponding assignments. This difference is mechanistically plausible because PCR selectively amplifies predefined targets, whereas shotgun sequencing distributes reads across all nucleic acid present in the extract. When target abundance is low and host/environmental background is high, the effective proportion of sequencing devoted to the pathogen can become extremely small [38,39,40,41]. CP17 illustrates the additional problem of taxonomic ambiguity: 81.6% of read pairs initially assigned to SARS-CoV-2 were removed as human-derived sequences after alignment-based host filtering, leaving 39 high-confidence read pairs (78 individual reads) and only 8.83% genome breadth.

Benchmarking studies in other low-titre, high-background matrices likewise show that untargeted metagenomics can yield too few pathogen reads for robust taxonomic or genome-level interpretation, while targeted amplification or capture can improve recovery [38,41,42,43]. Hybrid-capture and related enrichment approaches have produced large increases in viral read proportion and genome coverage in suitable settings [38,41,44,45,46,47]. These studies support the general principle that the target-to-background ratio is a major determinant of metagenomic performance. In the present Riyadh surface dataset, substantial host-derived and environmental sequence background consumed most of the available sequencing space, while target-virus assignments remained sparse. The data therefore motivate testing enrichment and optimised host reduction prospectively rather than assuming that additional untargeted depth alone would resolve the problem.

A major limitation is the absence of sequenced extraction blanks, library/no-template controls, and defined positive spike controls. This prevents direct attribution of the substantial background assignments and independent characterisation of the sequencing workflow’s target-recovery limit. The PCR-negative assignments documented in Section 3.3 could reflect the reagent background, environmental background, index-related artefacts, database/taxonomic ambiguity, or genuine target material below the PCR panel’s call threshold; however, the present design cannot distinguish among these explanations. This limitation is particularly important in a low-biomass matrix and is carried explicitly into the interpretation below.

### 4.2. The “Indoor City” Factor: Riyadh as a Representative Challenging Environment

Riyadh’s built environment functions as an “indoor city”: people spend most of their time in air-conditioned interiors, repeatedly contacting the same surfaces, while intensive cleaning regimes are used to maintain hygiene. Indoor microbiome work has shown that high-touch surfaces accumulate substantial human-associated material—skin cells, secretions, and human-derived microbes—so human DNA typically dominates low-biomass indoor samples [48,49,50]. At the same time, frequent cleaning and disinfection reduce the overall microbial biomass on high-touch surfaces, often lowering total bacterial and viral loads to near or below the limit of detection of culture-independent methods [48,49,50,51,52]. Reviews of cleaning indoor microbiomes emphasise that routine disinfection can substantially change surface communities and, in some settings, reduce abundance to the point where recolonization dynamics, rather than stable biomass, define the microbiome [50,52].

Riyadh’s combination of high indoor occupancy, repeated human contact, and intensive surface hygiene creates conditions that may be particularly challenging for shotgun viromics: continued deposition of host-associated nucleic acid occurs alongside cleaning that can reduce microbial biomass. Prior benchmarking identifies low-virus, high-background matrices as difficult for untargeted metagenomic recovery [38,39,40,41,42]. The sparse viral assignments observed here are therefore compatible with the known challenges of sequencing low-biomass, host-dominated surface material, although the present study cannot isolate the contribution of cleaning, host background, archival handling, or library preparation.

### 4.3. Recommendations: Target Enrichment as a Future Strategy

Shotgun metagenomics provided broader taxonomic context beyond the targeted panel, including assignments to commensal and environmental taxa and to off-panel viral taxa. Because sequenced negative controls and orthogonal validation were limited, these broader assignments are interpreted descriptively rather than as independently confirmed organism detections. This breadth remains an advantage of untargeted sequencing, but the same background dominated the available sequence space. Viral assignments comprised only 0.16% of classified read pairs, and the three respiratory RNA-virus taxa represented among the PCR-positive calls accounted for just 105 read pairs across all ten pools. Target recovery was variable and could not be explained by Ct value or host fraction alone: unrecovered PCR-positive events spanned Ct 33.9–37.9, recovered events Ct 33.9–38.3, and the highest viral count (adenovirus in HP10, 53 read pairs) arose from the highest Ct in the selected dataset (38.3). A low target abundance, abundant host/microbial background, archival handling, library preparation, and the absence of enrichment may all have contributed. With only 14 evaluable PCR-positive events and no sequenced negative controls, the study cannot attribute the pattern to a single mechanism. Targeted PCR and untargeted sequencing are therefore complementary rather than interchangeable in this dataset: the former provides targeted amplification of defined panel targets, while the latter provides broader taxonomic and discovery context.

The combined evidence suggests that plain, unenriched shotgun sequencing may require methodological augmentation for robust viral target recovery from low-biomass, host-dominated high-touch surfaces. Target enrichment is one plausible strategy. Prior studies show that hybrid-capture or related enrichment approaches can markedly increase viral read proportions and improve genome coverage in low-titre, high host backgrounds [38,41,42,44,45,46,47,53,54,55]. These literature findings support testing enrichment prospectively in this matrix, but the present study did not evaluate an enrichment workflow and therefore does not establish how much improvement would occur in these samples.

For future work in Riyadh and comparable built environments, a stronger design would combine optimised preprocessing/host reduction, viral enrichment where appropriate, sequenced extraction and library blanks, defined positive spike controls, and prospective parallel processing of PCR and sequencing from the same extract. Such a design would preserve the discovery advantage of sequencing while allowing a fairer assessment of target recovery, background contamination, and genome-level performance.

### 4.4. Study Limitations

This study has several important limitations. First, the ten sequenced pools were retrospectively selected after PCR screening because each contained at least one viral PCR-positive event. The study therefore cannot estimate diagnostic sensitivity or specificity; the 10/14 result is positive agreement within a preselected cohort, with substantial uncertainty (exact 95% CI, 41.9–91.6%). Second, no sequenced extraction blanks, library/no-template controls, or defined positive spike controls were included, limiting attribution of the extensive PCR-negative taxonomic assignments and preventing independent characterisation of the sequencing workflow’s target-recovery performance. Third, the ≥1 read-pair threshold is intentionally permissive. Section 3.3 shows that 37.0% of evaluable PCR-negative cells received a target assignment at this threshold, and CP17 demonstrates that k-mer classification can include human-derived misassignments. We therefore performed post hoc reference alignment for all ten PCR+/mNGS+ target-pool cells. This secondary analysis found predominantly full-length support for four assignments, only short partial support for two, and no alignment to the selected representative reference for four sparse genus-level assignments (Supplementary Table S7). Because the genus-level targets were checked against single representative references and no independent wet-lab confirmation was performed, this analysis cannot adjudicate the true status of every assignment. Accordingly, the primary agreement analysis remains as taxonomic assignment concordance rather than verified organism detection, and genus/clade assignments were not upgraded to species-level confirmation without appropriate orthogonal support. Fourth, rRNA depletion was used but no experimental host-genomic-DNA depletion was performed, leaving substantial host-derived sequence background. Fifth, the two methods were applied to archived material at different points in its storage history. Samples were collected between February 2023 and May 2024 and stored at −80 °C; PCR screening was performed in January–March 2025, whereas aliquots later sent for sequencing underwent an additional freeze–thaw cycle and international shipment before extraction/library preparation approximately four months later. The VTM manufacturer cautions that repeated freeze–thaw can reduce recovery of viable organisms, and additional archival handling may also affect sparse nucleic-acid templates. This asymmetry means the comparison was not a same-extract, same-time experiment and may have contributed to unrecovered viral PCR-positive events. The available bulk RNA-integrity data, however, do not show an obvious association between overall RNA integrity and viral target recovery. RQN values measured at library preparation were moderate to good across all ten pools (6.1–8.6; median 7.35). The mean RQN was similar in pools containing an unrecovered viral event (7.33) and pools in which viral targets were recovered (7.44); CP20, tied for the highest RQN in the dataset at 8.6, contained one of the unrecovered viral events. Thus, the available bulk RNA-integrity measurements did not show an obvious association with viral target recovery, although degradation of low-abundance viral RNA during archival handling cannot be excluded. Finally, the study represents ten archived pools from a specific arid urban surface-surveillance cohort. Broader performance conclusions require prospective studies that include PCR-negative environmental samples, parallel same-extract processing, sequenced controls, enrichment comparisons, and orthogonal target confirmation.

## 5. Conclusions

This retrospective exploratory comparison found that unenriched shotgun metagenomics produced corresponding taxonomic assignments for only part of a preselected PCR-positive target set and simultaneously generated substantial target assignment in PCR-negative pool-by-target cells. At the permissive ≥ 1 read-pair threshold, positive agreement was 71.4% (10/14; exact 95% CI, 41.9–91.6%), while background assignment was 37.0%; at ≥10 read pairs, agreement fell to 35.7% and background remained 24.8%. These results should not be interpreted as diagnostic sensitivity/specificity estimates or as proof that one platform is universally superior. Instead, they show the limitations of using low-count, unenriched k-mer taxonomic assignments as a stand-alone pathogen call in this low-biomass, host-dominated retrospective dataset.

Post hoc reference alignment further showed that the apparently concordant assignments were heterogeneous: four had predominantly full-length support against the selected reference, two had only short partial support, and four sparse genus-level assignments had no support against the chosen representative reference. Duplicate-aware summarization also reduced several raw pair counts to substantially fewer distinct alignment spans, reinforcing the need to interpret sparse k-mer assignments cautiously.

Metagenomics nevertheless provided broader taxonomic context that the targeted panel could not, including descriptive assignments to commensal, environmental and off-panel viral taxa; these broader assignments were not independently validated as organism detections. For the specific task of recovering the previously reported low-template panel targets, however, multiplex PCR yielded more targeted positive calls than the unenriched shotgun workflow, while sequencing assignments remained affected by background and sparse genome support. Future prospective comparisons should process both methods in parallel from the same extract and include sequenced negative controls, positive spike controls, orthogonal reference alignment and, ideally, an enrichment arm. Under these conditions, the relative roles of targeted PCR and metagenomic sequencing in high-touch-surface surveillance can be evaluated more rigorously.

## Figures and Tables

**Table 1:** Domain-level composition of classified read pairs across the 10 pools.

Domain	Classified Read Pairs	Share of Classified
Bacteria	50,426,022	97.6%
Eukaryota	1,085,442	2.1%
Viruses	84,183	0.16%
Archaea	48,801	0.09%
(Unclassified)	21,100,500	— no database match

**Table 2:** Off-panel and non-target taxa, with their most likely origin and interpretation.

Taxon (Off-Panel)	Likely/Possible Context	Interpretation
*Cutibacterium acnes*; *Malassezia restricta*; *Corynebacterium*; *Streptococcus*	Human skin/oral commensals	Surface microbiome; expected contact flora
*Cutibacterium/Propionibacterium* phages	Skin bacteriophages	Track bacterial hosts; not a disease signal
Beta-papillomavirus/HPV-5	Human skin virome	Commensal, non-pathogenic; off-panel
Adeno-associated virus 2/dependoparvovirus	Common commensal/contaminant	Non-pathogenic; off-panel
Tobamoviruses (tomato brown rugose, tobacco mosaic)	Plant viruses	Environmental/dietary markers; not human
*Aspergillus*/*Penicillium*; *Saccharomyces cerevisiae*	Environmental moulds/food yeast	Environmental provenance markers
*Plasmodium relictum*	Avian malaria parasite/possible cross-reactive assignment	Not interpreted as a human-pathogen detection; reported with caution
Parainfluenza virus 5 (simian virus 5)	Possible reagent- or database-associated signal	Not interpreted as a confirmed environmental pathogen detection

## Data Availability

The original raw metagenomic sequencing datasets contain human host reads and have not been publicly released in this form; they are available from the corresponding author on reasonable request subject to appropriate data-handling safeguards. Host-depleted sequencing reads used for downstream taxonomic analysis have been deposited in the NCBI Sequence Read Archive under BioProject accession PRJNA1532818. Processed host-depleted taxonomic read counts and the complete PCR–metagenomics comparison are provided in Supplementary Table S1; per-pool host-removal metrics in Supplementary Table S4; taxonomic composition in Supplementary Table S5; CP17 reference-alignment evidence, including per-position depth and distinct mate-pair alignment spans, in Supplementary Table S6; and the post hoc reference-alignment and duplicate-aware summary for all ten PCR+/mNGS+ target-pool combinations in Supplementary Table S7. The targeted PCR comparator data were originally reported in the companion study [33]. The custom taxon-to-QIAstat mapping script, target mapping file, presence/absence and heatmap workflow, and deduplication/breadth-analysis scripts are publicly available at https://github.com/malhamdoosh/SurfaceMetagenomicScripts (accessed on 22 September 2026).

## Supplementary Materials

The following supporting information can be downloaded at: https://www.mdpi.com/article/10.3390/microorganisms14102233/s1, Supplementary Document S1: Sequencing quality summary report (Azenta NGS Data Report and MultiQC/fastp summary); Supplementary Table S1: Complete pool-by-target comparison of QIAstat-Dx PCR and shotgun-metagenomic taxonomic assignment, including PCR calls, Ct, aggregated Kraken2 read pairs, evaluability, category at ≥1/≥5/≥10 read-pair thresholds, per-target background-assignment rates, and threshold analysis; Supplementary Table S2: Sample-level PCR panel results and collection metadata from the previously published surveillance dataset [33]; Supplementary Table S3: Pool composition, PCR screening results, and Ct values for the 19 positive pools, reproduced from the previously published dataset [33] for comparator documentation; Supplementary Table S4: Per-pool sequencing and host-removal metrics (source data for Figure 1); Supplementary Table S5: Domain-level and viral-fraction taxonomic composition (source data for Figure 4); Supplementary Table S6: SARS-CoV-2 alignment summary, cluster table, per-position depth, and distinct mate-pair alignment-span inventory for pool CP17 (source data for Figure 3); Supplementary Table S7: Reference-alignment and duplicate-aware re-analysis of all ten PCR+/mNGS+ target-pool combinations at the ≥1 read-pair threshold, including representative references, mapped-read support, distinct mate-pair alignment spans, singleton loci, and reference breadth.
