# Supplementary material for: Complementary Use of Shotgun Metagenomics and Multiplex PCR for Pathogen Detection on High-Touch Surfaces
Source: Microorganisms. 2026 Oct 3;14(10):2233. doi: 10.3390/microorganisms14102233 (PMC13707448; doi:10.3390/microorganisms14102233)
Supplement: Supplementary file 1 [file microorganisms-14-02233-s001.zip › microorganisms-4496583-supplementary/Supplementary_Document_S1.pdf]

Supplementary File S6. Sequencing Quality Summary Report

This file summarizes the sequencing quality and per-sample read metrics for the 10 pooled high-touch surface samples sequenced in this study (Azenta project 30-1192731958; Illumina NovaSeq X Plus, 25B flow cell, 2×150 bp). It has two parts:

Part A — Provider sequencing statistics (Azenta)

Per-sample raw read counts, yield (Mbases), mean quality score, and percentage of bases ≥ Q30, together with the overall run statistics.

Part B — Pre- and post-trimming quality control (MultiQC / fastp)

Aggregated per-sample quality metrics including reads passing filter, adapter content, duplication, GC content, sequence quality before and after fastp trimming, insert-size distribution, and the fraction of reads classified as human (Homo sapiens) versus microbial/unclassified. Trimming: fastp v0.24.0; host removal: bwa-mem2 v2.2.1 against GRCh38; taxonomic classification: Kraken2 v2.17.1 (database k2_pluspf_20250714).

Note: read counts are reported as sequenced by the provider; Kraken2 processes paired reads, so classification counts are expressed in read pairs elsewhere in the manuscript. The per-pool host fraction plotted in Figure 1 and tabulated in Supplementary Table S4 is derived from bwa-mem2 alignment to GRCh38 and is methodologically distinct from the Kraken2-based "% Homo sapiens" column reported below.

AZENTA LIFE SCIENCES

Project Summary Sample Sequencing Statistics Overall Sequencing Statistics FAQs

AZENTA NGS DATA REPORT

1. PROJECT SUMMARY

Customer	Mohamad Taisir Ghiba
Email	443105998@student.ksu.edu.sa
Sample	•
QuoteNumber	30-1192731958
Configuration	Illumina 2x150bp

2. SAMPLE SEQUENCING STATISTICS

Show 10 entries Search:

Table 2.1 Sample Sequencing Statistics

Project	Sample ID	Barcode Sequence	# Reads	Yield (Mbases)	Mean Quality Score	% Bases >= 30
30-1192731958	CP18	ACATACAC+AGCACAAG	19,183,830	5,755	38.50	92.75
30-1192731958	AP5	TATCAGTA+TTACTACT	18,520,878	5,556	38.61	93.25
30-1192731958	AP9	TTTCCATC+TCCGAATG	18,745,092	5,624	37.92	90.00
30-1192731958	HP10	CAAGAAGT+CGACTAGG	18,836,098	5,651	38.60	93.20
30-1192731958	HP6	ACACGTAT+TTACATCG	18,393,883	5,518	38.65	93.42
30-1192731958	AP7	CGAGTCAG+CCTTAGTA	19,538,103	5,862	38.54	92.93
30-1192731958	HP2	GCTTAAGT+TGTTTACT	19,096,697	5,729	38.52	92.90
30-1192731958	CP8	CCAATGAA+TAGCTCAC	24,730,059	7,419	36.87	84.87
30-1192731958	CP17	ATCAGTTG+CCCGCGTT	18,833,688	5,650	38.32	91.86
30-1192731958	CP20	CTCCATAT+GGGTAAGC	20,709,688	6,213	38.47	92.52

Select Columns Download

Showing 1 to 10 of 10 entries

Previous 1 Next

3. OVERALL SEQUENCING STATISTICS

Table 3.1 Overall Sequencing Statistics

Project	# Reads	Yield (Mbases)	Mean Quality Score	% Bases >= 30
30-1192731958	196,588,016	58,977	38.26	91.57

4. FAQS

If you have any further questions or concerns, please consider examining our FAQ page located here.

Azenta Life Sciences
Next Generation Sequencing
Email: ngs@azenta.com
Phone: 908-222-0711 ext 1

MultiQC

A modular tool to aggregate results from bioinformatics analyses across many samples into a single report.

Report generated on 2025-11-25, 09:56 +03 based on data in: `/ssr/analysis/Virology_Ghiba/processed_data`

General Statistics

Copy table | Configure Columns | Plot | Showing $^{20}/_{20}$ rows and $^{8}/_{10}$ columns.

Sample Name	% Homo sapiens	% Top 5 Species	% Unclassified	% Duplication	M Reads After Filtering	GC content	% PF	% Adapter
AP5_R1_001				31.0%	35.2	53.5%	94.9%	20.6%
AP5_krak[illegible]report	41.0%	53.9%	8.0%					
AP7_R1_001				34.8%	36.5	52.5%	93.5%	24.0%
AP7_krak[illegible]report	39.6%	51.6%	8.7%					
AP9_R1_001				42.9%	32.3	53.0%	86.1%	24.2%
AP9_krak[illegible]2_report	22.7%	27.5%	50.7%					
CP17_R1_001				34.1%	35.2	57.9%	93.3%	17.3%
CP17_kral[illegible]n2_repor	26.0%	27.3%	24.2%					
CP18_R1_001				23.9%	36.7	50.7%	95.8%	17.3%
CP18_kral[illegible]repor	51.3%	53.1%	23.6%					
CP20_R1_001				36.3%	38.9	53.9%	93.9%	12.6%
CP20_kral[illegible]n2_repor	22.8%	33.7%	14.6%					
CP8_R1_001				46.8%	33.5	56.8%	67.7%	32.4%
CP8_krak[illegible]2_report	15.3%	21.8%	37.6%					
HP10_R1_001				28.3%	35.9	53.7%	95.2%	19.2%
HP10_kral[illegible]repor	45.3%	54.8%	6.6%					
HP2_R1_001				32.8%	36.0	50.5%	94.2%	20.3%
HP2_krak[illegible]port	60.9%	64.3%	7.2%					
HP6_R1_001				35.6%	34.7	47.5%	94.2%	19.4%

Sample Name	% Homo sapiens	% Top 5 Species	% Unclassified	% Duplication	M Reads After Filtering	GC content	% PF	% Adapter
HP6_krak[illegible]port	66.8%	69.3%	5.1%					

Kraken

Kraken is a taxonomic classification tool that uses exact k-mer matches to find the lowest common ancestor (LCA) of a given sequence. *DOI: 10.1186/gb-2014-15-3-r46.*

Top taxa

The number of reads falling into the top 5 taxa across different ranks.

Counts | Percentages

Species | Genus | Family | Order | Class | Phylum | Kingdom | Domain | Root

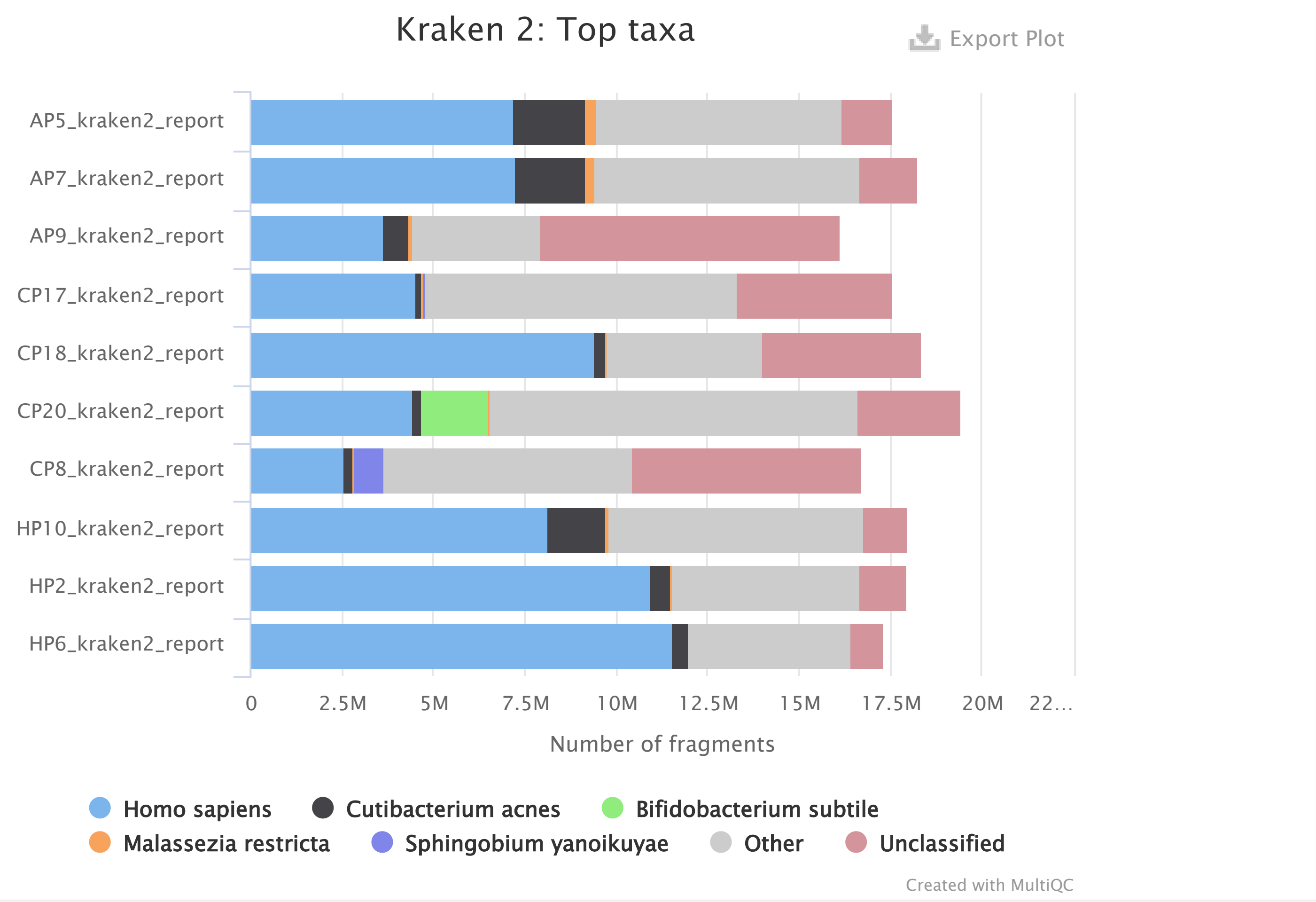

fastp

fastp An ultra-fast all-in-one FASTQ preprocessor (QC, adapters, trimming, filtering, splitting...). *DOI: 10.1093/bioinformatics/bty560.*

Filtered Reads

Filtering statistics of sampled reads.

Number of Reads | Percentages

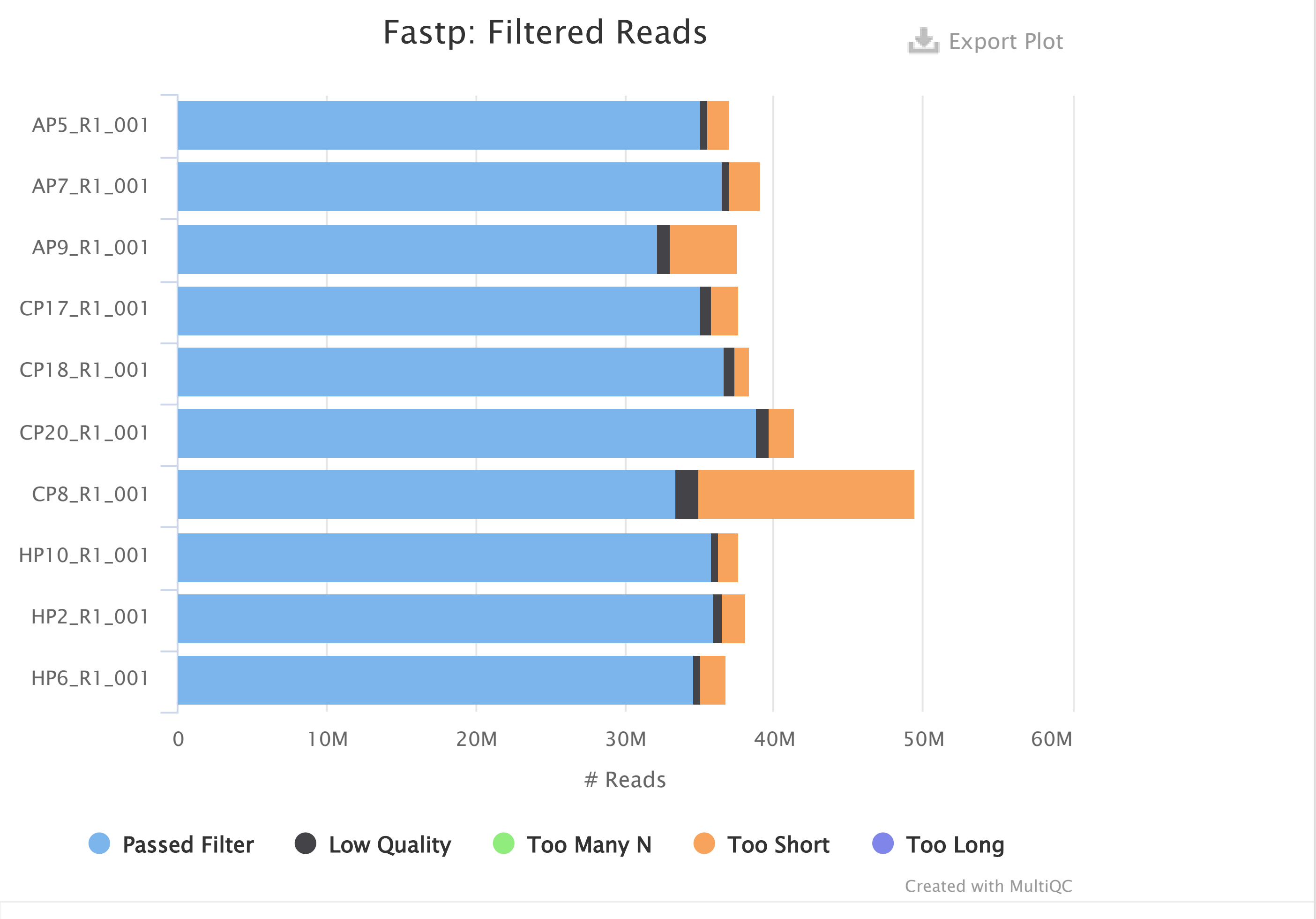

Insert Sizes

Insert size estimation of sampled reads.

Y-Limits: on

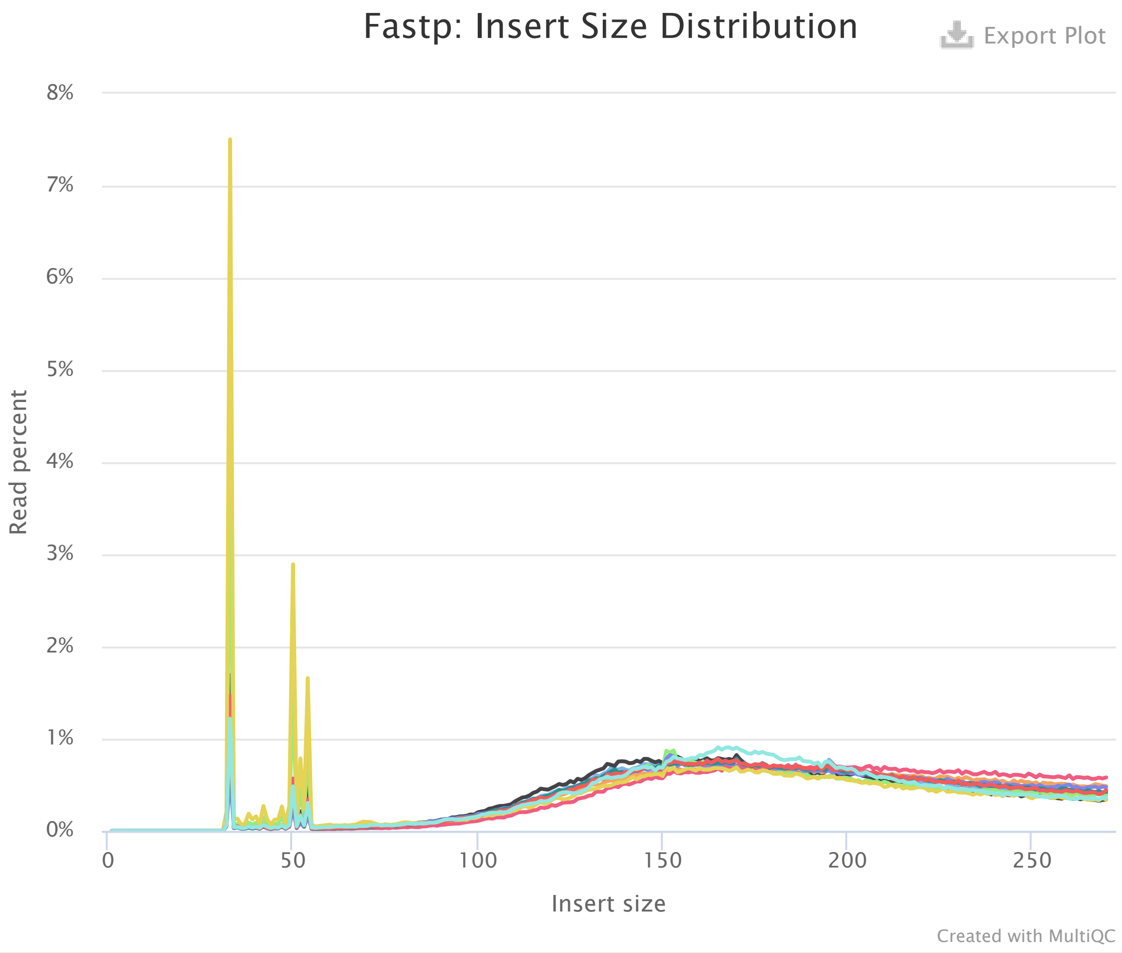

Sequence Quality

Average sequencing quality over each base of all reads.

Y-Limits: on

Read 1: Before filtering	Read 1: After filtering	Read 2: Before filtering	Read 2: After filtering

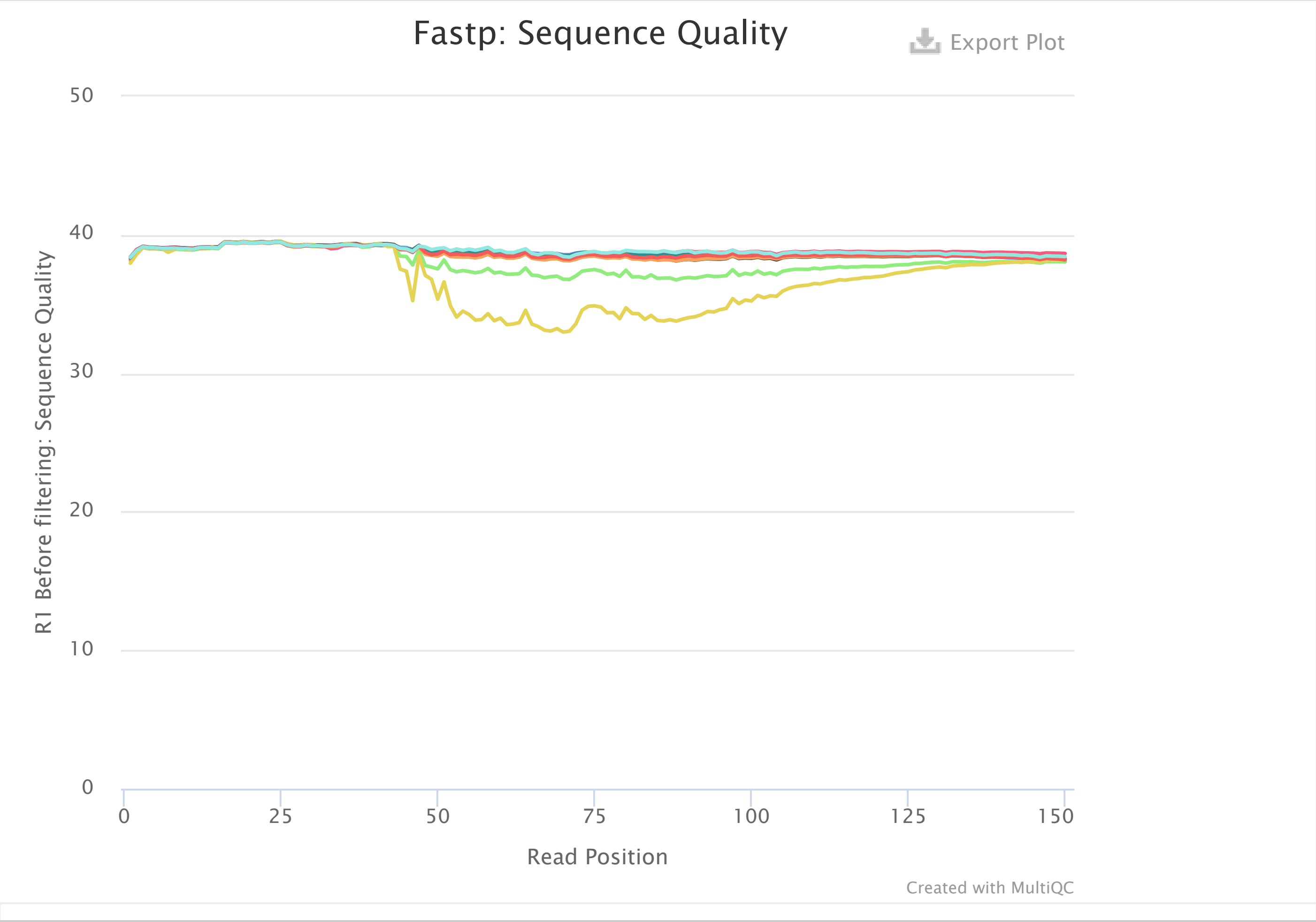

GC Content

Average GC content over each base of all reads.

Y-Limits: on

Read 1: Before filtering | Read 1: After filtering | Read 2: Before filtering | Read 2: After filtering

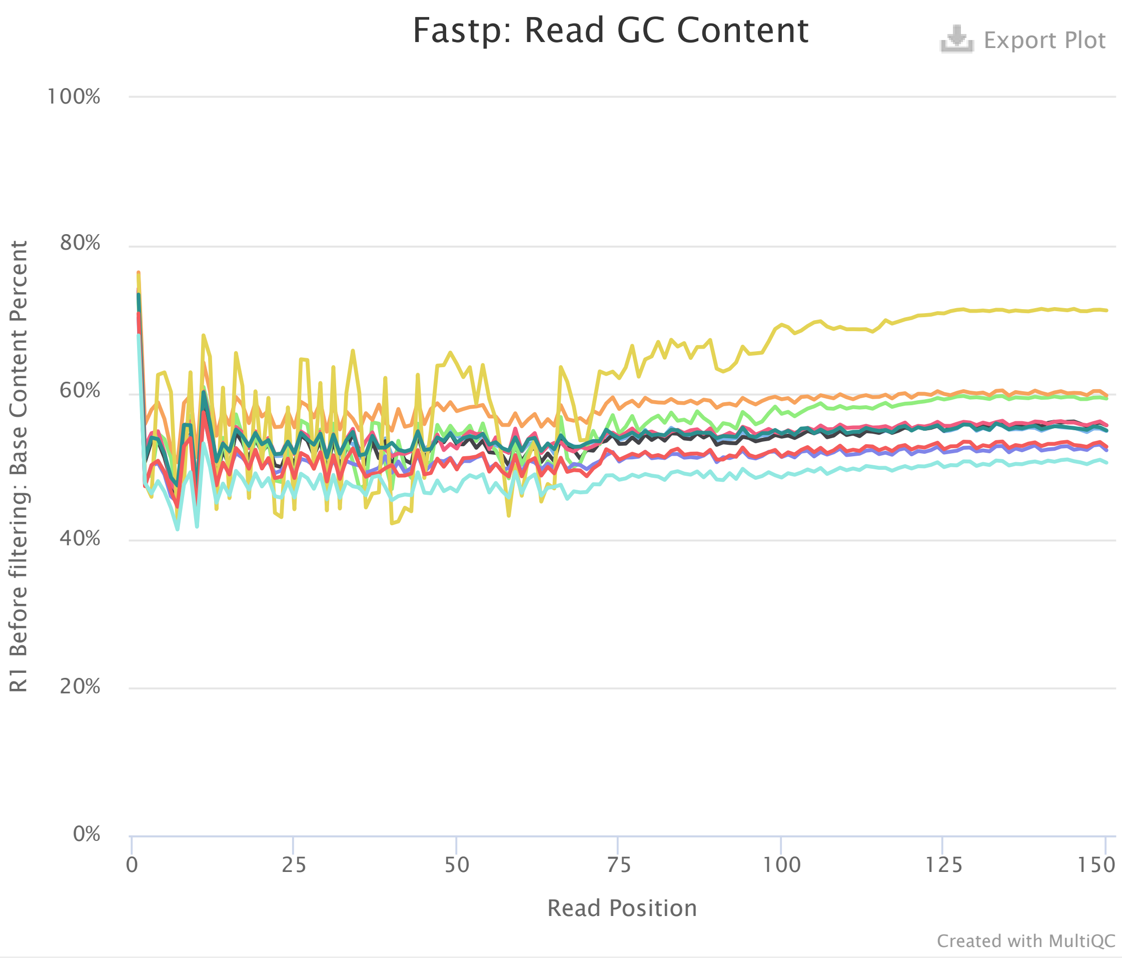

N content

Average N content over each base of all reads.

Y-Limits: on

Read 1: Before filtering | Read 1: After filtering | Read 2: Before filtering | Read 2: After filtering

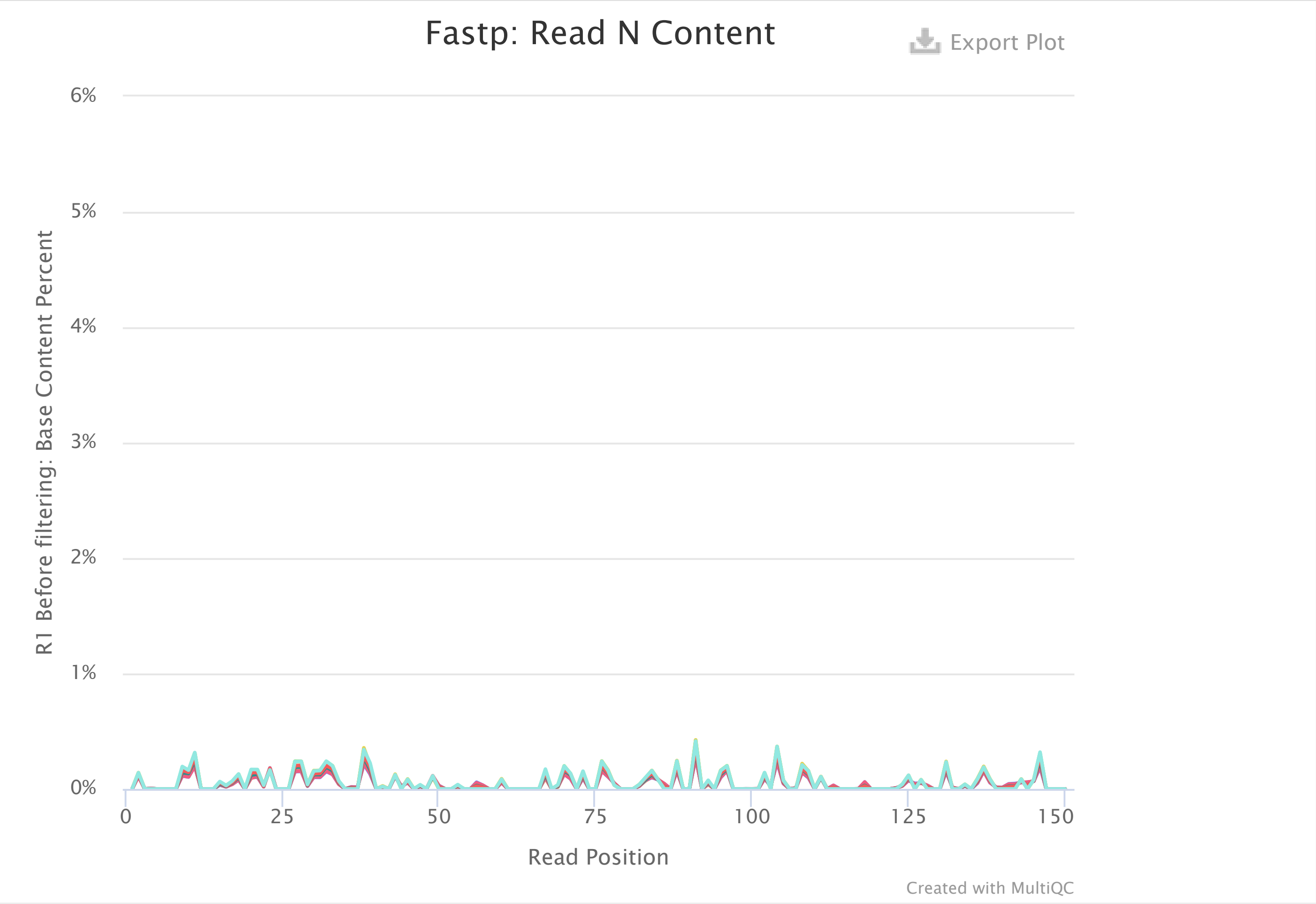

MultiQC v1.14 - Written by Phil Ewels, available on GitHub.
This report uses HighCharts, jQuery, jQuery UI, Bootstrap, FileSaver.js and clipboard.js.
